# Efficacy and safety of different immunotherapies combined with chemotherapy as first-line therapy in patients with small cell lung cancer: a network meta-analysis

**DOI:** 10.3389/fimmu.2024.1362537

**Published:** 2024-04-17

**Authors:** Siyao Gong, Qian Li, Xin Yu, Sha Yang

**Affiliations:** College of Acupuncture and Massage, Chengdu University of Traditional Chinese Medicine, Chengdu, China

**Keywords:** immunotherapies, chemotherapy, small-cell lung cancer, network meta-analysis, serplulimab

## Abstract

**Background:**

The efficacy and safety of different immunosuppressants combined with chemotherapy in treating patients with small-cell lung cancer (extensive-disease small-cell lung cancer, limited-disease small-cell lung cancer and relapsed small-cell lung cancer) are still unknown, and there are no reports directly comparing the efficacy and safety of other immunotherapies.

**Objective:**

This study aimed to compare the efficacy and safety of first-line immunotherapy combined with chemotherapy in patients with small-cell lung cancer.

**Method:**

We searched Pubmed, Embase, Cochrane Library, CNKI, and Wanfang databases for relevant articles published from inception to November 11, 2020. The risk of bias of the included studies was conducted using the Cochrane risk-of-bias (RoB) tool. Multiple Bayesian network meta-analyses were performed. They conducted data analysis using R Studio and STATA version 15.1. The outcomes comprised overall survival (OS), progression-free survival (PFS), stability of response (SOR), duration of response (DOR) and adverse events of grade 3 or higher (AE grade≥3). A 95% confidence interval (CI) was provided for each estimate.

**Results:**

This meta-analysis included 16 RCT studies with 5898 patients. For OS, relative to chemotherapy (MD=-4.49; 95%CI [-7.97, -1.03]), durvalumab plus tremelimumab (MD=-4.62; 95%CI [-9.08, -0.11]), ipilimumab (MD=-4.26; 95%CI [-8.01, -0.3]) and nivolumab(MD=-5.66; 95%CI [-10.44, -1.11]) and nivolumab plus ipilimumab (MD=-4.56; 95%CI [-8.7, -0.1]), serplulimab can significantly increase the OS of SCLC patients. There was no significant difference between PFS, SOR and DOR. Analysis of AE showed that different immunotherapy combined chemotherapy regimens were similar to single chemotherapy regarding the overall incidence of AE grade≥3. However, after the cumulative ranking of the common symptoms of different adverse reactions, it was found that nivolumab ranked first in the occurrence probability of anemia (99.08%), fatigue (84.78%), and decreased appetite (89.66%). durvalumab was the most likely in nausea (75.4%). Pembrolizumab (76.24%) was most likely to cause pruritus. Chemotherapy combined with immunotherapy caused less diarrhea than chemotherapy alone (80.16%).

**Conclusions:**

According to our analysis, serplulimab combined with chemotherapy is more likely to show better efficacy with a manageable safety profile for small-cell lung cancer. However, the evidence for this comparison shows some limitations due to the number of literature.

**Systematic review registration:**

https://www.crd.york.ac.uk/PROSPERO/, identifier CRD42023486053.

## Introduction

Small cell lung cancer (SCLC), which accounts for 15-17% of all lung cancers, is the most malignant type of lung cancer and is prone to recurrence (1), and has been designated as an orphan disease by the European Medicines Agency (EMA) (2). SCLC is characterized by rapid proliferation, uncomplicated metastasis, rapid drug resistance and poor prognosis. Most patients have systemic metastasis at diagnosis, and only 5%-15% have no symptoms in the early stage (3). Even though the incidence of SCLC has declined in the past years mainly due to a decrease in smoking habits, the prognosis of this malignancy remains dismal, with an overall 5-year survival rate of 7% (4–6). According to the American Veterans Lung Cancer Association, there are two classifications: limited-disease small cell lung cancer (LD-SCLC) and extensive-disease small cell lung cancer (ED-SCLC), and limited-stage usually indicates that the lung cancer may be confined to one side of the lung tissue. In the extensive stage, diffusion occurs more often, resulting in metastasis in many body parts (7). ED-SCLC accounts for about 70% of SCLC. And many neurologic and endocrine paraneoplastic syndromes are associated with SCLC (8).

In the last few decades, SCLC, in addition to surgical treatment and platinum-etoposide chemotherapy combination, has remained the backbone of the frontline therapy of ED-SCLC, with the addition of concurrent radiotherapy for LD-SCLC (4, 9), no more effective breakthroughs were found (10). Most SCLC patients initially respond well to standard chemotherapy. Still, with the development of chemotherapy resistance, the prognosis of patients is significantly affected, and patients are prone to relapse after chemotherapy (4). In line with non-small cell lung cancer (NSCLC), immune checkpoint inhibitors (ICIs) debuted in the treatment algorithm and enriched the available pharmacological weaponry (11), creating a new field of SCLC treatment (12). Immunotherapeutic agents for small-cell lung cancer among programmed cell death 1 (PD-1), P.D. ligand 1 (PD-L1), and cytotoxic T lymphocyte-associated protein (CTLA-4) inhibitors et al., some immune checkpoint inhibitors (ICIs), such as serplulimab, atezolizumab, pembrolizumab et al. has been proven to improve overall survival (OS) and progression-free survival (PFS) of SCLC patients, and has fewer side effects compared with chemotherapy (13–15). In the landmark IMpower133 study, 201 patients were randomly assigned to the atezolizumab group and 202 patients to the placebo group. At a median follow-up of 13.9 months, the median OS was 12.3 months in the atezolizumab group and 10.3 months in the placebo group. The median PFS was 5.2 months and 4.3 months, respectively. The safety profile of atezolizumab plus carboplatin and etoposide was consistent with the previously reported safety profile of the individual agents, with no new findings observed (13). In a CASPIAN phase III study, durvalumab combined with chemotherapy had more prolonged overall survival (median OS was 12.9) compared with chemotherapy alone, and the efficacy of durvalumab was maintained with longer follow-up time (36-month OS rate was 17.6%) (16, 17). But, adding tremelimumab to durvalumab did not significantly improve the outcome (17). In addition, clinical research results show that chemotherapy plus ICI cannot prolong OS or PFS versus chemotherapy alone in patients with SCLC (15, 18).

Although several meta-analyses have provided good evidence for first-line ICIs for the treatment of ED-SCLC (19–21), until now, direct/indirect comparisons between global immunotherapy drugs for SCLC have been lacking; therefore, we conducted a Bayesian network meta-analysis to compare the efficacy and safety of ICIs combined with chemotherapy for SCLC. This provides more effective evidence for clinicians to develop more effective treatment options.

## Methods

We performed a systematic review and NMA according to the Preferred Reporting Items for Systematic Reviews and meta-analyses (22). This study protocol has been registered on the International Prospective Register of Systematic Reviews (PROSPERO) (CRD42023486053).

### Search strategy

With the help of a professional librarian, we searched Pubmed, Embase, Cochrane Library, CNKI, and Wanfang databases for relevant articles published from inception to November 11, 2023. The search terms were combined with MeSH words and free words to comprehensively search all SCLC-related randomized controlled trials (RCTs). In addition, we reviewed International Agency for Research on Cancer (IARC) monographs and references cited in identified articles. The search terms included the following keywords: small cell lung carcinoma, PD-L1, CTLA-4, ipilimumab, atezolizumab, durvalumab, pembrolizumab, adebrelimab, serplulimab, nivolumab, randomized clinical trial, and their related MeSH terms. The detailed strategy is shown in **Supplementary Table S1**.

### Selection and eligibility criteria

The inclusion criteria were as follows:

(1)Patients older than 18 years of age were eligible for small-cell lung cancer confirmed histologically or cytologically.
(2)The treatment group was based on chemotherapy combined with immunotherapy or alone (ipilimumab, atezolizumab, durvalumab, pembrolizumab, adebrelimab, serplulimab, durvalumab+tremelimumab, nivolumab, ipilimumab+nivolumab).
(3)The main outcome was overall survival.
(4)Prospective, randomized, controlled clinical trials.

The exclusion criteria were as follows:

(1)Duplicate published literature, conference abstracts, animal studies, and retrospective studies.
(2)Outcome indicators did not meet the inclusion criteria of RCTs.
(3)RCT with active brain metastases.
(4)RCT of phase I study.
(5)The full text of the study could not be downloaded.

Two researchers (Qian Li and Xin Yu) initially screened the literature retrieved by reading the titles and abstracts imported into the endnote, and when the abstracts were insufficient to determine whether the study met the inclusion or exclusion criteria, conducted a full-text review to evaluate the studies ultimately included in the analysis. Reviews, clinical guidelines, and conference abstracts over the last three years were thoroughly checked to ensure the integrity of the included studies. At the same time, the two authors did all the data extraction. The two researchers completed the process independently, and any problems encountered could be discussed with another researcher (Siyao Gong). When no suitable data was available, we contacted the authors of the relevant studies.

### Data extraction

For the final included studies, basic information should be extracted, including title, study name, study phase, first author, year of publication, sample size, age, gender, disease type, smoking status, diagnostic criteria, inclusion criteria, exclusion criteria, intervention measures, PD-L1 expression, and Eastern Cooperative Oncology Group (ECOG) performance status score, brain metastases, race, liver metastases. The clinical outcomes extracted included a median with corresponding 95% confidence intervals (95% CIs) for OS, (randomization to death regardless of any causes) and PFS (randomization to the progression of any causes or death irrespective of any causes). The duration of response or stable response was assessed in patients who had an objective confirmed response and was defined as the time from the first occurrence of a documented objective response to the time of disease progression as determined or death from any cause, whichever occurred first. And incidence of grade ≥3 adverse events in each immunotherapy combination. And assessment of risk bias.

### Risk of bias assessment

According to the Cochrane Handbook for Systematic Reviews of Interventions (23), through the Cochrane RoB2 tools for bias risk assessment randomized controlled trial. The tool has five different fields for generating the entire RoB. Each domain was evaluated with one of the following options: “Low RoB”, “Some Concerns”, and “High RoB”. Following the individual domain assessment, we categorized studies with just 1 out of 5 risk domains with a “Some Concerns” judgement as a “Low RoB”. Studies with one “High RoB” domain were judged as “High RoB”.

### Statistical analysis

Network meta-analyses (NMA) were performed using the “gemtc” packages based on the R statistical environment. Given the heterogeneity between the trials, a Bayesian Hierarchical Random Effects Model was fitted for multiple comparisons of different treatments for SCLC. Based on the Bayesian framework of the Monte Carlo Markov Chain (MCMC) consistency model, a posteriori distribution of the queried nodes was studied by setting 50000 iterations and 20000 annealing with a step size of 1. The probability ranking diagrams of therapeutic effect were established by the “Rank. Probability” function. At the same time, we ranked the likelihood of different treatment options based on cumulative ranking probabilities (SUCRAs). For dichotomous variables (AE, SOR), OR and 95%CI were reported. Continuous variables (OS, PFS, DOR) were reported as M.D. and 95%CI. The node-splitting method was adopted to check the local inconsistency, and a local inconsistency was considered between two interventions when a p-value was less than 0.05. In addition, we used Stata 15.1 software to conduct funnel plots to detect whether there was publication bias in different articles.

## Results

### Systematic review and characteristics

**Figure 1:**
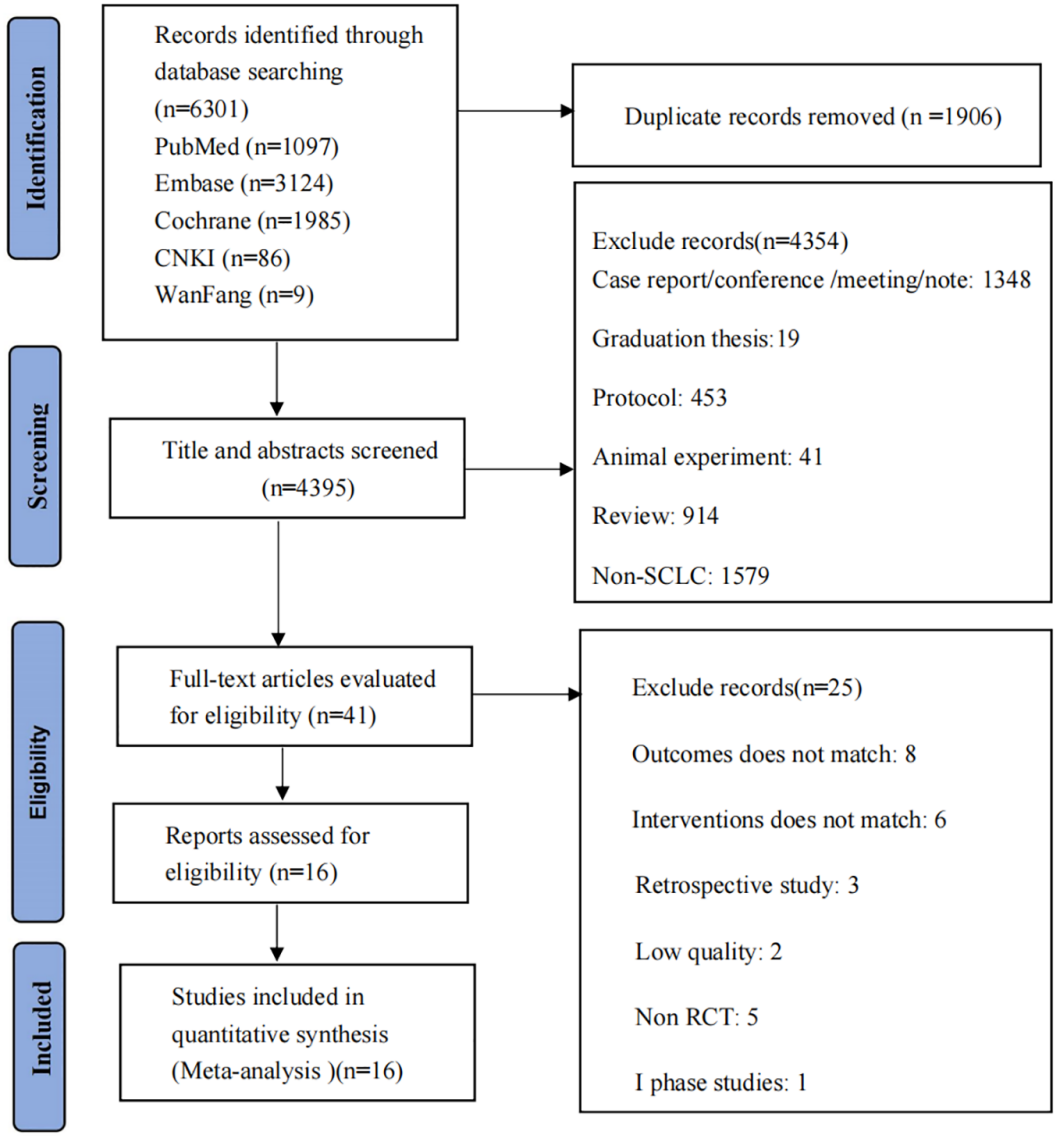
Flow diagram of studies identified, included and excluded.

A comprehensive literature search identified a total of 6301 articles, including Pubmed (n=1097), Embase (n=3124), Cochrane Library (n=1985), CNKI (n=86) and Wanfang (n=9). After reading the title and abstract, 41 articles met our inclusion criteria. After reading the full text, 25 articles were excluded, including five non-RCT studies, eight studies with inappropriate outcome measurement, six with inappropriate intervention, three retrospective studies, two low-quality literature, and one as a phase I study. Finally, there were 16 studies for this meta-analysis, including 12 subjects, as shown in **Figure 1**.

**Figure 2:**
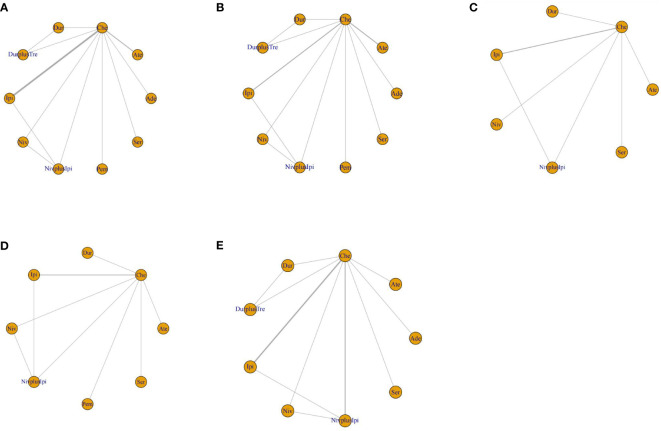
Network diagrams of comparisons on various treatments with SCLC. **(A)** OS; **(B)** PFS; **(C)** DOR; **(D)** SOR; **(E)** AE grade ≥ 3. Che, chemotherapy; Ate, Atezolizumab; Dur, Durvalumab; DurplusTre, Durvalumab + Tremelimumab; Ipi, Ipilimumab; Pem, Pembrolizumab; Ser, Serplulimab; Niv, Nivolumab; NivplusIpi, Nivolumab + Ipilimumab; Ade, Adebrelimab. PFS, progression-free survival; OS, overall survival; DOR, duration of response; SOR, stable of response; AE grade ≥ 3, adverse events of grade ≥ 3.

The meta-analysis included 5898 patients, of whom 3917 were known to be male. Among them, eight studies were ED-SCLC, one was LD-SCLC, two were relapsed SCLC (RE-SCLC), and one was SCLC. The included literature was mainly published between 2013 and 2022 in English. Phase III studies accounted for 66.67% of all studies. Most diagnostic Criteria were Response Evaluation Criteria in Solid Tumors (RECIST) and version 1.1 (71.42%). There were also significant differences in the interventions included in the studies. atezolizumab was used in two studies, ipilimumab combined with nivolumab in three studies, nivolumab alone in one study, ipilimumab alone in two studies, and serplulimab in one study. One study involved pembrolizumab, one involved adebrelimab, and one involved durvalumab. **Figure 2** and **Supplementary Figure S1** show the network plots, and the information on each enrolled study is listed in **Table 1** and **Supplementary Table S1**.

**Table 1:** Baseline characteristics of trials.

Study ID	Type of disease	Study name	Phase	Tretment	No. of pations(males)	Age	Tumor assessment criteria	OS(HR;95%CI)	PFS(HR;95%CI)
Reck M, et al. 2013 (24)	ED-SCLC	CA184-041	II	Concurrent Ipi+PC	43	NR	mWHO/irRC	0.95(0.59-1.54)	NR
				Phased Ipi+PC	42	NR		0.75(0.46-1.23)	NR
				PC	45	NR			
Goldman JW, et al., 2021; Paz-Ares L, et al. 2019-2022 (16, 17, 25)	ED-SCLC	CASPIAN	III	Dur+Tre+EP	268(190)	63(58-68)	RECIST V.1.1	0.81(0.67-0.97)	0.84(0.70-1.01)
				Dur+EP	268(202)	62(58-68)		0.71(0.60-0.86)	0.80(0.66-0.96)
				EP	269(184)	63(57-68)			
Pujol JL, et al. 2019 (26)	SCLC	IFCT-1603	II	Ate	49(30)	63.5(51.1-85.5)	RECIST V.1.1	0.84(0.45-1.58)	2.26(1.30-3.93)
				Che	24(13)	65.9(51.8-81.0)			
Wang J, et al. 2022 (19)	ED-SCLC	CAPSTONE-1	III	Ade+Che	230(184)	62(55–66)	RECIST V.1.1	0.72(0.58-0.90)	0.67(0.54-0.83)
				Pla+Che	232(188)	62(56–67)			
Peters S, et al. 2022 (27)	LD-SCLC	CheckMate-032	II	Niv+Ipi+Che	78(50)	61.1(37.7-83.2)	TNM	0.94(0.59-1.50)	1.01(0.65-1.55)
				Che	75(42)	61.9(38.6-77.3)			
Cheng Y, et al. 2022 (14)	ED-SCLC	ASTRUM-005	III	Ser+Che	389(317)	63(28-76)	RECIST V.1.1	0.63(0.49-0.82)	0.48(0.38-0.59)
				Pla+Che	196(164)	62(31-83)			
Rudin CM, et al. 2020 (15)	ED-SCLC	KEYNOTE-604	III	Pem+EP	228(152)	64(24-81)	RECIST V.1.1	0.80(0.64-0.98)	0.75(0.61-0.91)
				Pla+EP	225(142)	65(37-83)			
Owonikoko TK, et al. 2021 (28)	ED-SCLC	CheckMate 451	III	Niv+Ipi+Che	279(181)	64.0(39-85)	ECOG PS	0.92(0.75-1.12)	0.72(0.60-0.87)
				Ipi+Che	280(177)	65.0(32-84)		0.84(0.69-1.02)	0.67(0.56-0.81)
				Pla+Che	275(175)	64.0(44-84)			
Reck M, et al. 2016 (18)	ED-SCLC	CA184-156	III	Ipi+Che	478(317)	62(39-85)	ECOG PS	0.94(0.81-1.09)	0.85(0.75-0.97)
				Pla+Che	476(326)	63(36-81)			
Spigel DR, et al. 2021 (29)	RE-SCLC	CheckMate 331	III	Niv+TA	284(174)	62(37–85)	RECIST V.1.1	0.86(0.72-1.04)	1.41(1.18-1.69)
				Che+TA	285(177)	61(34–82)			
Antonia SJ, et al. 2019 (30)	RE-SCLC	CheckMate 032	II	Niv+Ipi	61(35)	66(58–71)	RECIST V.1.1	NR	NR
				Niv	98(61)	63(57–68)			
Horn L, et al. 2018-2022 (13)	ED-SCLC	IMpower133	III	Ate+EC	201(129)	64(28–90)	RECIST V.1.1	0.70(0.54-0.91)	0.77(0.62-0.96)
				Pla+EC	202(132)	64(26–87)			

LD-SCLC, limit-disease small cell lung cancer; ED-SCLC, extensive-disease small cell lung cancer; RE-SCLC, recurrent small cell lung cancer; HR, hazard ratio; NR, not reported; OS, overall survival; PFS, progression-free survival; RECIST, Response Evaluation Criteria in Solid Tumors; mWHO, modified World Health Organization criteria; irRC, newly proposed immune-related response criteria; TNM, tumour-node-metastasis; ECOG PS, Eastern Cooperative Oncology Group performance status; PC, paclitaxel/carboplatin; Ipi, Ipilimumab; SC, cisplatin/carboplatin; Dur, Durvalumab; Che, chemotherapy; Pla, Placebo; EC, etoposide/carboplatin; Niv, Nivolumab; EP, Platinum/etoposide; Tre,Tremelimumab; Pem, Pembrolizumab; Ser, Serplulimab; Ate, Atezolizumab; TA, topotecan/amrubicin.

There was a low risk of intervention bias, missing outcome data bias, and selection bias in outcome measures, except that six studies did not mention hidden methods for randomization, and three studies had possible outcome selective reporting bias. The overall risk bias of all studies was “low risk”, and the assessment of the risk of bias is shown in **Supplementary Figure S2**.

### Network meta-analyses for outcomes

**Figure 3:**
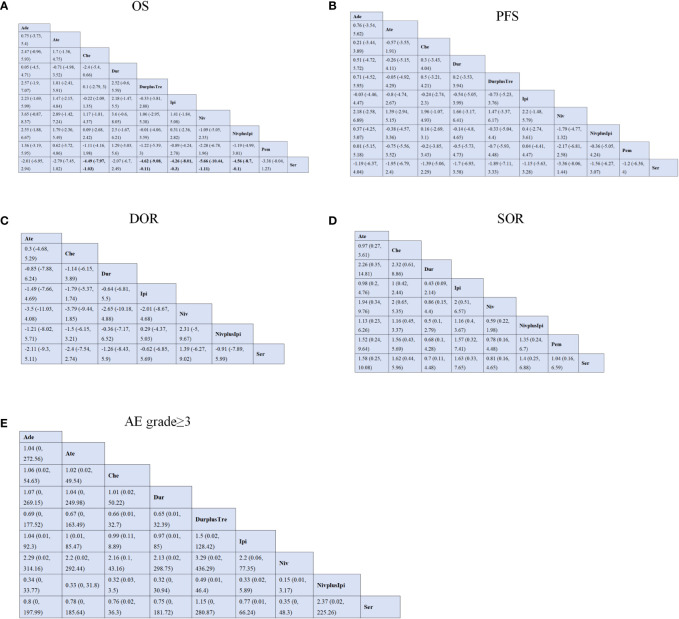
Efficacy and safety profiles of the Bayesian network meta-analysis in patients with SCLC. **(A)** OS; **(B)** PFS; **(C)** DOR; **(D)** SOR; **(E)** AE grade ≥ 3. Che, chemotherapy; Ate, Atezolizumab; Dur, Durvalumab; DurplusTre, Durvalumab + Tremelimumab; Ipi, Ipilimumab; Pem, Pembrolizumab; Ser, Serplulimab; Niv, Nivolumab; NivplusIpi, Nivolumab + Ipilimumab; PFS, progression-free survival; Ade, Adebrelimab. OS, overall survival; DOR, duration of response; SOR, stable of response; AE grade ≥ 3, adverse events of grade ≥ 3.

In this network analysis, 11 studies (13–15, 18, 24, 26, 28) evaluated OS, including 10 treatments. From the NMA results in **Figure 3A**, compared with chemotherapy (MD=-4.49; 95%CI [-7.97, -1.03]), durvalumab plus tremelimumab (MD=-4.62; 95%CI [-9.08, -0.11]), ipilimumab (MD=-4.26; 95%CI [-8.01, -0.3]), nivolumab (MD=-5.66; 95%CI [-10.44, -1.11]) and nivolumab plus ipilimumab (MD=-4.56; 95%CI [-8.7, -0.1]), chemotherapy plus serplulimab can significantly prolonged the OS in patients with SCLC (**Figure 3A**, **Supplementary Figure S3A**).

Regarding PFS, ten studies (13–15, 17, 18, 26, 28, 29, 31, 32) contain ten regimens to observe the changes in SCLC patients in PFS. Our NMA found no statistical differences between different treatment regimens. According to the results in **Figure 3B**, serplulimab showed better efficacy in comparison with nivolumab (MD=-3.36; 95%CI [-8.06, 1.44]). In addition, Compared with nivolumab, adebrelimab (MD=2.18; 95%CI [-2.58, 6.89]) and ipilimumab (MD=2.2, 95%CI [-1.48, 5.79])has considerable advantages in increasing PFS (**Figure 3B**, **Supplementary Figure S3B**).

Regarding the duration of response (DOR) of six studies (13, 14, 18, 28, 29, 31), there was no significant difference in efficacy among all regimens. Compared with nivolumab plus ipilimumab, nivolumab (MD=2.31, 95%CI [-5,9.67]) can improve the patient’s DOR. Interestingly, compared with serplulimab, nivolumab (MD=1.39, 95%CI [-6.27,9.02]) also helps to improve the DOR. However, compared with chemotherapy, atezolizumab(MD=0.3, 95%CI [-4.68,5,29]) was not more beneficial in raising DOR. Details are shown in **Figure 3C** and **Supplementary Figure S3C**.

For stable response (SOD), It compared eight treatments in eight studies (13–15, 18, 26, 28), and we found no statistical difference between the various regimens. Relative to nivolumab, ipilimumab (OR=2; 95%CI [0.51, 6.57]) increased SOR in patients. At the same time, compared with durvalumab, atezolizumab(OR=2.26; 95%CI [0.35, 14.81]) has the advantage of improving SOR. Ipilimumab, nivolumab, nivolumab plus ipilimumab, pembrolizumab and serplulimab all showed low efficacy compared with durvalumab. The details are shown in **Figure 3D** and **Supplementary Figure S3D**.

### Subgroup network meta-analysis for OS by brain metastasis

We collated the OS subgroups of all included studies, and only two studies (13, 14)analyzed the median OS of brain metastases, and the regimens had atezolizumab, serplulimab, and chemotherapy. We could not perform a network analysis because of the limited number of published articles. For SCLC patients with brain metastases, compared with chemotherapy (MD=9.85) and atezolizumab (MD=8.50), serplulimab (MD=13.9) showed significantly better efficacies. For patients without brain metastases, compared with atezolizumab (MD=12.60) and chemotherapy (MD=10.84), serplulimab (MD=15.6) was the most long-lasting regimen in OS rate.

**Figure 4:**
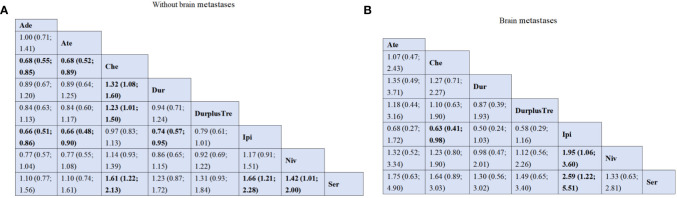
Efficacy and safety profiles of the Bayesian network meta-analysis on overall survival in patients with SCLC. **(A)** HRs and 95% CI for without brain metastases patients. **(B)** HRs and 95% CI for with brain metastases patients. Che, chemotherapy; Ate, Atezolizumab; Dur, Durvalumab; DurplusTre, Durvalumab + Tremelimumab; Ipi, Ipilimumab; Pem, Pembrolizumab; Ser, Serplulimab; Niv, Nivolumab; NivplusIpi, Nivolumab + Ipilimumab; Ade, Adebrelimab.

In addition, we conducted a network analysis of H.R. of patients with or without brain metastases in all included kinds of literature, which included six studies (13, 14, 16, 18, 29, 32) and eight treatments: serplulimab, atezolizumab, ipilimumab, nivolumab, adebrelimab, durvalumab plus tremelimumab, durvalumab and chemotherapy. For patients without brain metastases, adebrelimab (HR=0.68; 95%CI [0.55; 0.85]) and atezolizumab (HR=0.68; 95%CI [0.52; 0.89]) both showed better survival benefits, compared with chemotherapy. Adebrelimab(HR=0.66; 95%CI [0.51; 0.86]), atezolizumab (HR=0.66; 95%CI [0.48; 0.90]) and durvalumab (HR=0.74; 95%CI [0.57; 0.95]) all presented significantly better survival profit in comparison with ipilimumab. Compared with chemotherapy(HR=1.32; 95%CI [1.08; 1.60]), durvalumab provides better survival profit. Durvalumab plus tremelimumab also showed better OS than chemotherapy (HR=1.23; 95%CI [1.01; 1.50]). Serplulimab significantly increased OS in patients without brain metastases compared with chemotherapy (HR=1.61; 95%CI [1.22; 2.13]), ipilimumab (HR=1.66; 95%CI [1.21; 2.28])and nivolumab (HR=1.42; 95%CI [1.01; 2.00]). However, compared with Ipilimumab, chemotherapy (HR=0.63; 95%CI [0.41; 0.98]) showed better efficacy for patients with brain metastases. Compared with ipilimumab(HR=2.59; 95%CI [1.22; 5.51]), serplulimab showed a better survival effect. Meanwhile, compared with ipilimumab(HR=1.95; 95%CI [1.06; 3.60]), nivolumab also showed promising efficacy (**Figures 4A, B**).

### Network meta-analyses for AEs of grade≥3

The incidence of AE at grade 3 or above did not differ significantly between any two of the following regimens: adebrelimab, atezolizumab, durvalumab, durvalumab plus tremelimumab, ipilimumab, nivolumab, nivolumab plus ipilimumab, serplulimab. Meanwhile, adebrelimab (OR=1.06; 95%CI [0.02, 54.63]), atezolizumab (OR=1.02; 95%CI [0.02, 49.54]), durvalumab (OR=1.01; 95%CI [0.02, 50.22]) and ipilimumab (OR=0.99; 95%CI [0.11, 8.89]) was similar to the safety of conventional chemotherapy alone, OR close to 1. The details of the results are shown in **Figure 3E** and **Supplementary Figure S3E**.

### Rank probability

**Figure 5:**
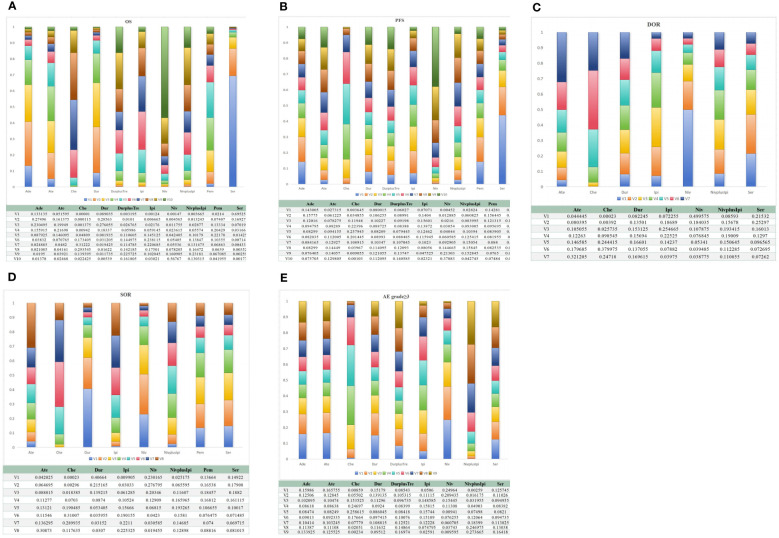
Bayesian ranking profiles for immunotherapy combinations on efficacy and safety for patients with SCLC. **(A)** OS; **(B)** PFS; **(C)** DOR; **(D)** SOR; **(E)** AE grade ≥ 3. Che, chemotherapy; Ate, Atezolizumab; Dur, Durvalumab; DurplusTre, Durvalumab + Tremelimumab; Ipi, Ipilimumab; Pem, Pembrolizumab; Ser, Serplulimab; Niv, Nivolumab; NivplusIpi, Nivolumab + Ipilimumab; Ade, Adebrelimab. PFS, progression-free survival; OS, overall survival; DOR, duration of response; SOR, stable of response; AE grade ≥ 3, adverse events of grade ≥ 3.

Bayesian ranking profiles showed the probability that each regimen had the best outcome and safety. Of all the first-line immunotherapies for SCLC patients, serplulimab had the highest probability (94.02%; 79.40%) of ranking first for better OS and PFS. Interestingly, nivolumab seemed associated with the highest possibility of ranking first for DOR and AE grade≥3 (78.77%;71.26%). In addition, Durvalumab (77.81%) ranks first in the cumulative probability ranking of SDR (**Figures 5A–E**, **Table 2**).

**Table 2:** Surface under the cumulative ranking (SUCRA) value on various treatments with SCLC.

	Durvalumab	Atezolizumab	Nivolumab	Pembrolizumab	Ipilimumab	Durvalumab+Tremelimumab	Chemotherapy	Nivolumab+Ipilimumab	Serplulimab	Adebrelimab
OS	75.37%	63.84%	20.17%	54.52%	35.90%	28.81%	30.13%	22.21%	94.02%	75.00%
PFS	46.47%	39.50%	14.72%	57.16%	60.13%	41.86%	53.75%	49.55%	79.41%	57.45%
DOR	45.35%	32.16%	78.76%	-	57.10%	-	21.47%	50.95%	64.20%	-
SOR	77.81%	33.40%	73.22%	58.44%	29.29%	-	26.77%	40.81%	60.25%	-
Brain metastases	63.05%	38.35%	62.45%	-	12.52%	51.60%	42.08%	-	79.97%	-
Without brain metastases	55.66%	67.58%	39.56%	-	22.40%	48.27%	20.43%	-	77.33%	68.77%
AE grade≥3	53.73%	52.62%	71.25%	-	53.34%	43.23%	54.38%	23.10%	46.71%	51.61%
Decreased appetite	63.63%	2.64%	89.66%	86.11%	36.37%	43.53%	65.25%	18.02%	-	44.77%
Anemia	48.45%	66.45%	99.08%	37.48%	37.85%	43.75%	37.23%	42.02%	47.99%	39.69%
Diarrhea	72.17%	50.68%	0.04%	76.66%	41.06%	61.11%	80.16%	18.13%	-	-
Pruritus	68.33%	46.04%	12.18%	76.25%	42.63%	68.13%	66.43%	20.01%	-	-
Fatigue	70.79%	44.38%	84.78%	41.62%	17.68%	75.61%	50.11%	15.03%	-	-
Nausea	75.40%	73.99%	62.41%	59.88%	59.70%	53.87%	52.66%	49.33%	6.53%	6.24%

Che, chemotherapy; Ate, Atezolizumab; Dur, Durvalumab; DurplusTre, Durvalumab + Tremelimumab; Ipi, Ipilimumab; Pem, Pembrolizumab; Ser, Serplulimab; Niv, Nivolumab; NivplusIpi, Nivolumab + Ipilimumab; Ade, Adebrelimab. PFS, progression-free survival; OS, overall survival; DOR, duration of response; SOR, stable of response; AE grade ≥ 3, adverse events of grade ≥ 3.

In addition, after the cumulative ordering of common AE, nivolumab ranked first in the occurrence probability of anemia (99.08%), fatigue (84.78%) and decreased appetite (89.66%). durvalumab was the most likely candidate for nausea (75.4%). Pembrolizumab (76.24%) was most likely to cause pruritus. And diarrhea caused by chemotherapy combined with immunotherapy was lower than single chemotherapy (80.16%), as shown in **Table 2**. Other toxicity characteristics are shown in **Supplementary Table S3**.

### Cluster analysis

**Figure 6:**
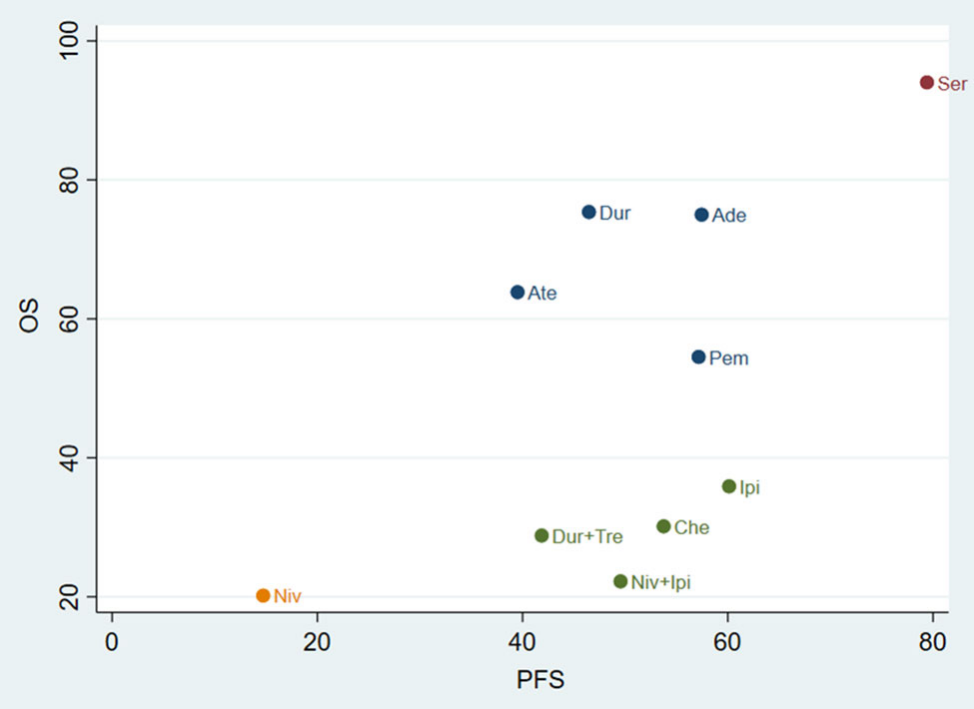
Cluster analysis plots for OS (y-axis) and PFS (x-axis). Atezolizumab; Dur, Durvalumab; DurplusTre, Durvalumab + Tremelimumab; Ipi, Ipilimumab; Pem, Pembrolizumab; Ser, Serplulimab; Niv, Nivolumab; NivplusIpi, Nivolumab + Ipilimumab; Ade, Adebrelimab. PFS, progression-free survival; OS, overall survival.

For the primary outcome measures of OS and PFS, we performed a cluster analysis to evaluate the best treatment for SCLC. We found that serplulimab was the best option for improving OS and PFS at the farthest point from the zero of the two-dimensional coordinates. In contrast, nivolumab had the worst overall ranking for the outcome measures tested. The details are shown in **Figure 6**.

### Publication bias and inconsistency analysis

We used STATA15.1 software to draw funnel plots to evaluate publication bias in the literature related to OS, PFS, DOR, SDR, and AE grade≥3. As shown in **Supplementary Figure S4**, visually, there was no publication bias in these studies. In addition, inconsistency test results also confirmed that the results of this study were relatively robust (**Supplementary Figure S5**).

## Discussion

This is the first complete meta-analysis to compare the relative efficacy of currently available first-line immunotherapy combinations for SCLC. This meta-analysis involved SD-SCLC, ED-SCLC, and RE-SCLC, which is more comprehensive than the previously published network meta-analysis (19, 20, 33, 34).In addition, this is the first network meta-analysis to analyze specific adverse effects of different ICIs. Our study helps clinicians to better decide the most appropriate use of different immune combination therapy regimens by fully considering the relevant survival benefits and toxicity characteristics of these clinical regimens in SCLC patients.

Our analysis showed that serplulimab, durvalumab, adebrelimab, atezolizumab, pembrolizumab, and ipilimumab combined with chemotherapy significantly prolonged overall survival. Among them, serplulimab had the best efficacy, similar to the results of the previous meta-analysis (35).

In terms of PFS, serplulimab, ipilimumab, adebrelimab, pembrolizumab, durvalumab, atezolizumab, nivolumab, nivolumab plus ipilimumab, durvalumab plus tremelimumab showed no statistically significant difference in efficacy compared with chemotherapy alone, a result similar to Mutlu’s meta-analysis (36). In addition, the CA184-156 was included in phase 3 randomized controlled study of 954 patients with SCLC (18); median progression-free survival was 4.6 months for chemotherapy plus ipilimumab versus 4.4 months for chemotherapy plus placebo (hazard ratio, 0.85; 95% CI, 0.75 to 0.97), There were no statistically significant differences between the two treatment groups (18). However, according to the cumulative probability ranking, serplulimab, ipilimumab, adebrelimab and pembrolizumab were more likely to prolong PFS in SCLC patients than chemotherapy alone.

Brain metastases represent an important clinical problem for patients with SCLC. However, the mechanisms underlying SCLC growth in the brain remain poorly understood. Mechanistically, the brain development factor Reelin, secreted by SCLC cells, recruits astrocytes to brain metastases (37). These astrocytes, in turn, promote SCLC growth by secreting neuronal pro-survival factors such as SERPINE1 (37).

Brain metastases at a single metastatic site are a good prognostic factor for SCLC patients (38). After subgroup analysis of OS, our NMA results show that serplulimab improves H.R. in patients with brain metastases. In SCLC patients without brain metastases, serplulimab, adebrelimab, and atezolizumab may be associated with longer-term survival benefits. However, we should strictly judge the results of the subgroup analysis because we lack a balanced background of patients.

The network analysis of DOR and SOR did not yield meaningful results. The results showed that immunotherapy monotherapy has similar efficacy compared with

Chemotherapy may be due to the limited number of research pieces of literature. However, after cumulative ranking, Serplulimab ranked third (60.3%) in DOR and second (64.2%) in SOR, indicating that serplulimab was beneficial for SCLC. Future clinical trials may elucidate the objective response of immunotherapy and chemotherapy strategies in patients with SCLC.

Immunosuppressive drugs combined with chemotherapy do not increase lethal toxicity in SCLC patients. This result is consistent with a published meta-analysis (19, 34). A meta-analysis has revealed a positive association between the occurrence of adverse events and improved treatment efficacy in patients treated with ICIs in lung cancer (39). It could explain the phenomenon that serplulimab ranked first in clinical efficacy but with relatively higher-graded AE.

Programmed cell death 1 (PD-1) is a transmembrane receptor, upon binding to its ligands PD-L1 (B7-H1) and PD-L2 (B7-DC) (40). PD1 is mainly expressed in activated T cells, B cells, macrophages and dendritic cells (40). Blocking PD1 can inhibit the function of T cells and down-regulate the immune response, thus opposing the activity of tumor cells, which is a essential critical immunotherapy strategy for SCLC. Serplulimab is a fully humanized PD1 monoclonal antibody. In Phase II clinical solid tumor study of Serplulimab, Serplulimab showed promising antitumor activity and a controllable safety profile regardless of the patient’s prior treatment (41). The ASTRUM-005 trial showed that the addition of serplulimab, a novel PD-1 inhibitor, to chemotherapy significantly improved OS by 4.5 months (15.4 months vs 10.9 months) and PFS (5.7 months vs 4.3 months) (14). In addition, Serplulimab was first approved in China in 2022 for treating advanced solid tumors, including small-cell lung cancer (42). In the *in vivo* models, serplulimab inhibited tumor growth at a lower dose than nivolumab (43). However, the mechanisms underlying the better survival outcome with serplulimab remain unknown, while the preclinical findings provide directions for future studies.

In addition, recent studies have found that anti-angiogenic drugs selectively inhibit active transcription in tumor cells and tumor-associated macrophages, enhance the immune response, and improve overall survival in patients with small-cell lung cancer (44).

### Limitations

Since most immunotherapy studies have only one published article, this may cause some bias in the accuracy of our results, and we will continue to monitor the publication of relevant articles to verify our existing results. Meanwhile, Chemotherapy methods and time points may differ among different studies, and various chemotherapy combinations may cause other synergistic effects. In this study, chemotherapy methods used in additional studies were uniformly treated, which may affect the robustness of the results. In addition, the results of this study may be affected by the lack of unpublished literature, and the efficacy results and conclusions of these immunotherapies need to be confirmed by more clinical studies.

## Conclusion

From our study, serplulimab combined with chemotherapy may be the best treatment to improve the survival of patients with SCLC (ED-SCLC, LD-SCLC and RE-SCLC) with manageable adverse effects, and durvalumab, adebrelimab, pembrolizumab, ipilimumab, atezolizumab also has some advantages. Clinicians can make reasonable choices based on the occurrence of adverse reactions and the effectiveness of different immunosuppressants.

## Data availability statement

The original contributions presented in the study are included in the article/**Supplementary Material**. Further inquiries can be directed to the corresponding authors.

## Author contributions

SG: Conceptualization, Formal analysis, Methodology, Software, Supervision, Validation, Writing – original draft, Writing – review & editing. QL: Data curation, Software, Investigation, Methodology, Validation, Visualization, Writing – original draft. XY: Data curation, Investigation, Methodology, Validation, Visualization, Writing – original draft, Conceptualization, Formal analysis, Supervision. SY: Project administration, Resources, Visualization, Writing – review & editing, Funding acquisition.
