# Supplementary material for: Efficacy and safety of different immunotherapies combined with chemotherapy as first-line therapy in patients with small cell lung cancer: a network meta-analysis
Source: Front Immunol. 2024 Apr 17;15:1362537. doi: 10.3389/fimmu.2024.1362537 (PMC11061408; doi:10.3389/fimmu.2024.1362537)
Supplement: Supplementary file 1 [file DataSheet_1.docx]

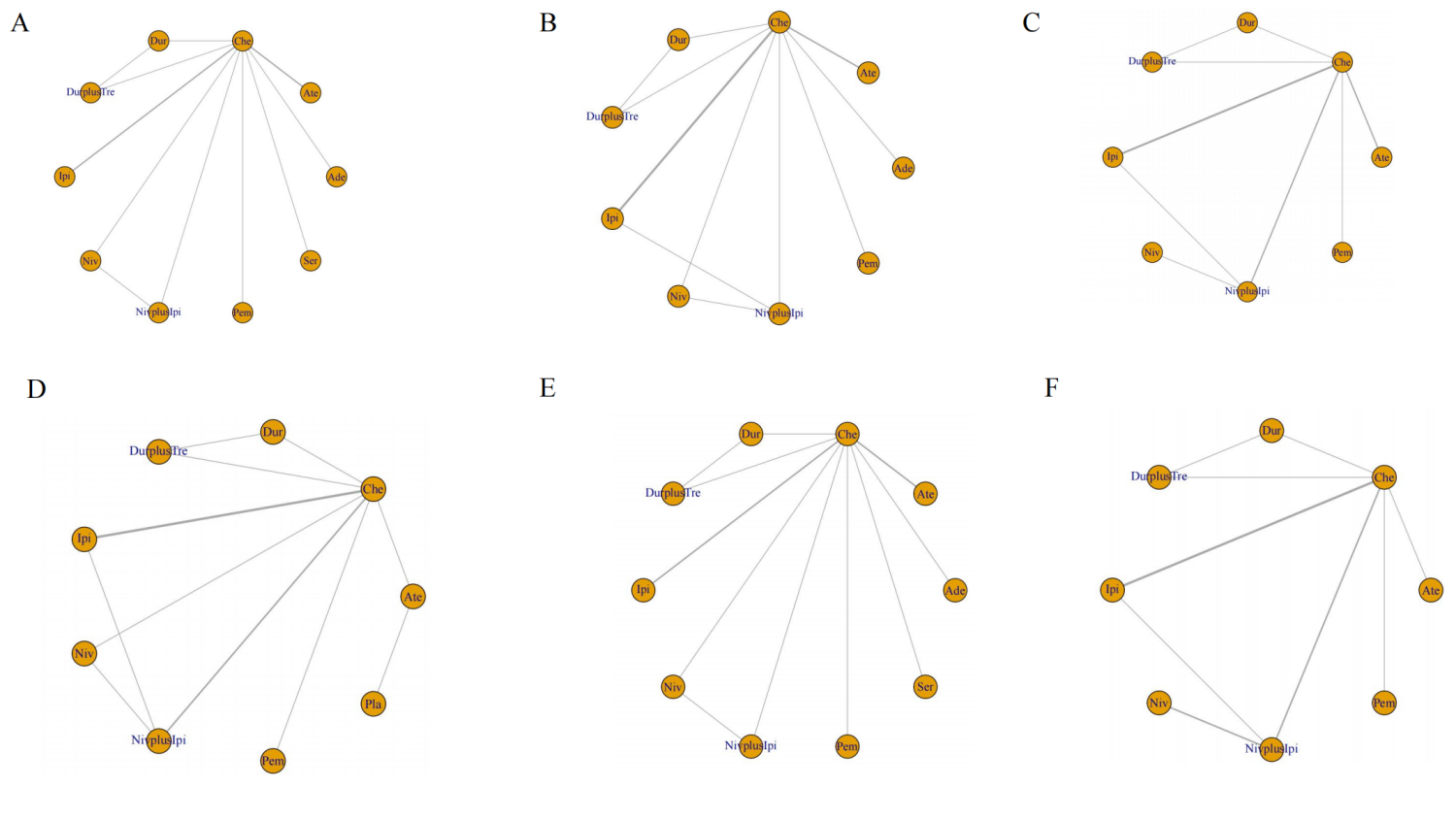


**Fiure S1.** Network diagrams of comparisons on various treatments for AE grade≥3. A: Anemia; B: Decreased appetite; C: Diarrhea; D: Fatigue; E: Nausea; F: Pruritus. Che: chemotherapy; Ate: Atezolizumab; Dur: Durvalumab; DurplusTre: Durvalumab + Tremelimumab; Ipi: Ipilimumab; Pem: Pembrolizumab; Ser: Serplulimab; Niv: Nivolumab; NivplusIpi: Nivolumab + Ipilimumab.


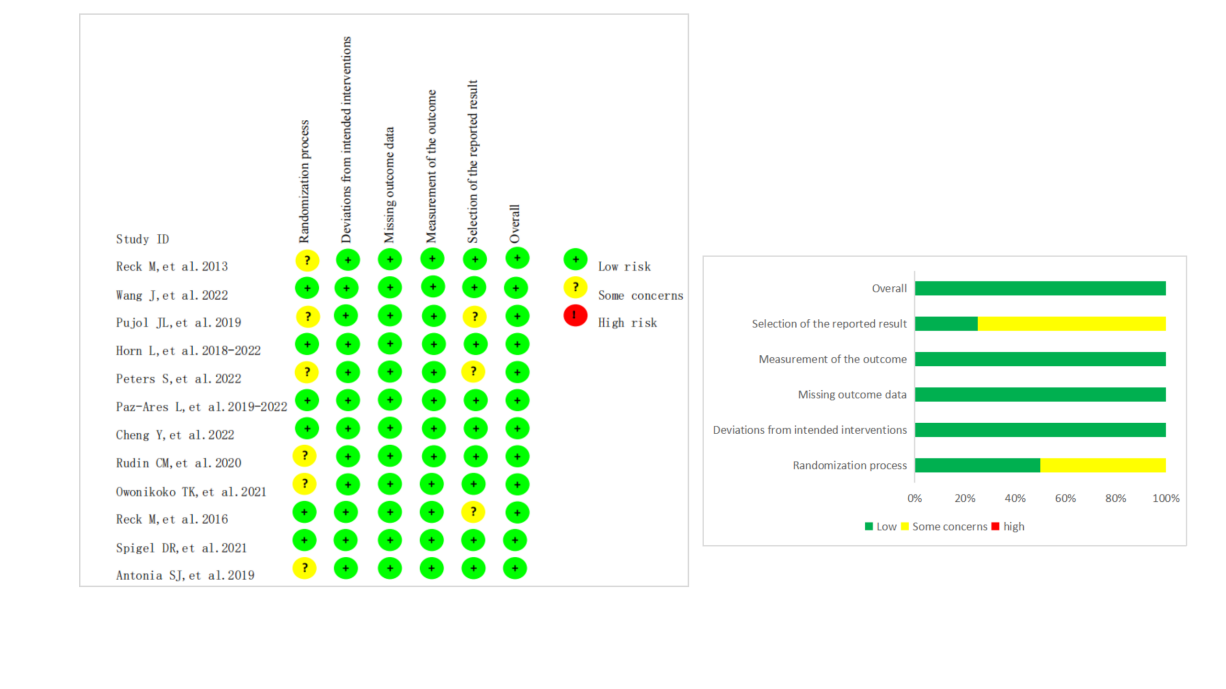


**Figure S2.** Assessment of risk bias.


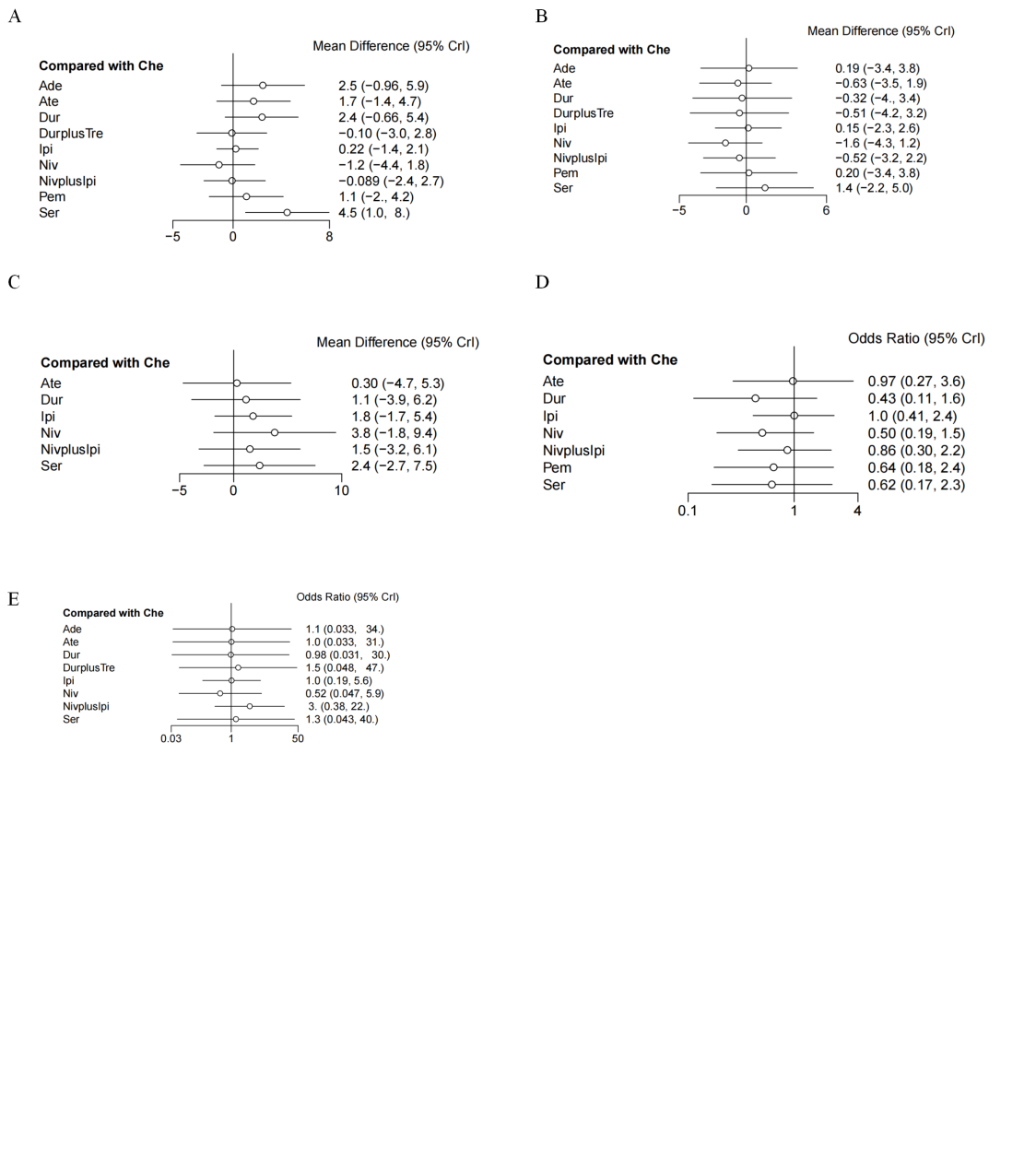


**Figure S3.** Forest plots of different interventions. A: OS; B: PFS; C: DOR; D: SOR; E: AE grade ≥ 3. Che: chemotherapy; Ate: Atezolizumab; Dur: Durvalumab; DurplusTre: Durvalumab + Tremelimumab; Ipi: Ipilimumab; Pem: Pembrolizumab; Ser: Serplulimab; Niv: Nivolumab; NivplusIpi: Nivolumab + Ipilimumab; Ade: Adebrelimab. PFS: progression-free survival; OS: overall survival; DOR: duration of response; SOR: stable of response; AE grade ≥ 3: adverse events of grade ≥ 3.


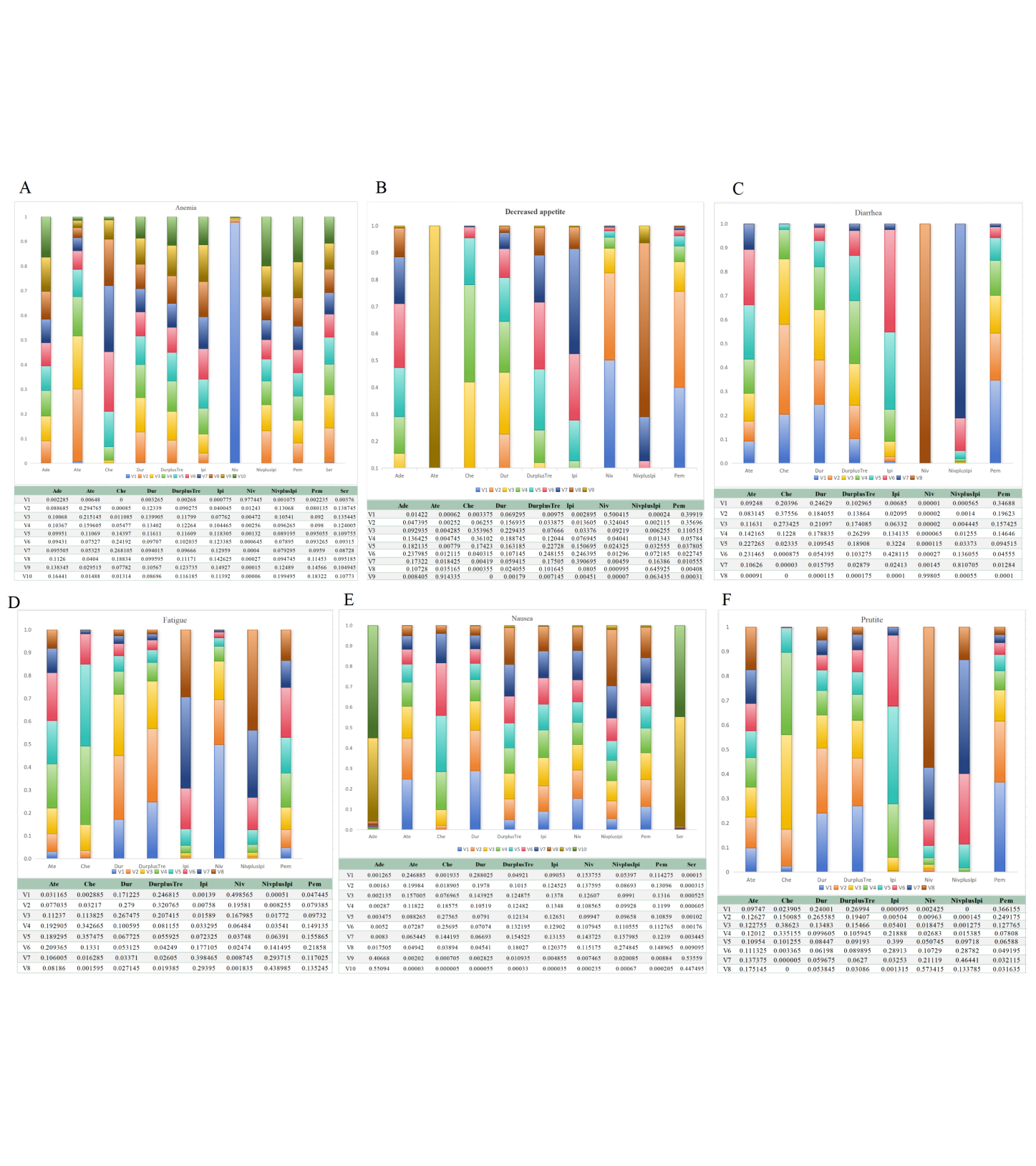


**Figure S4.** Bayesian ranking profiles for immunotherapy combinations on AE grade≥3. A: Anemia; B: Decreased appetite; C: Diarrhea; D: Fatigue; E: Nausea; F: Pruritus. Che: chemotherapy; Ate: Atezolizumab; Dur: Durvalumab; DurplusTre: Durvalumab + Tremelimumab; Ipi: Ipilimumab; Pem: Pembrolizumab; Ser: Serplulimab; Niv: Nivolumab; NivplusIpi: Nivolumab + Ipilimumab; Ade: Adebrelimab.

**
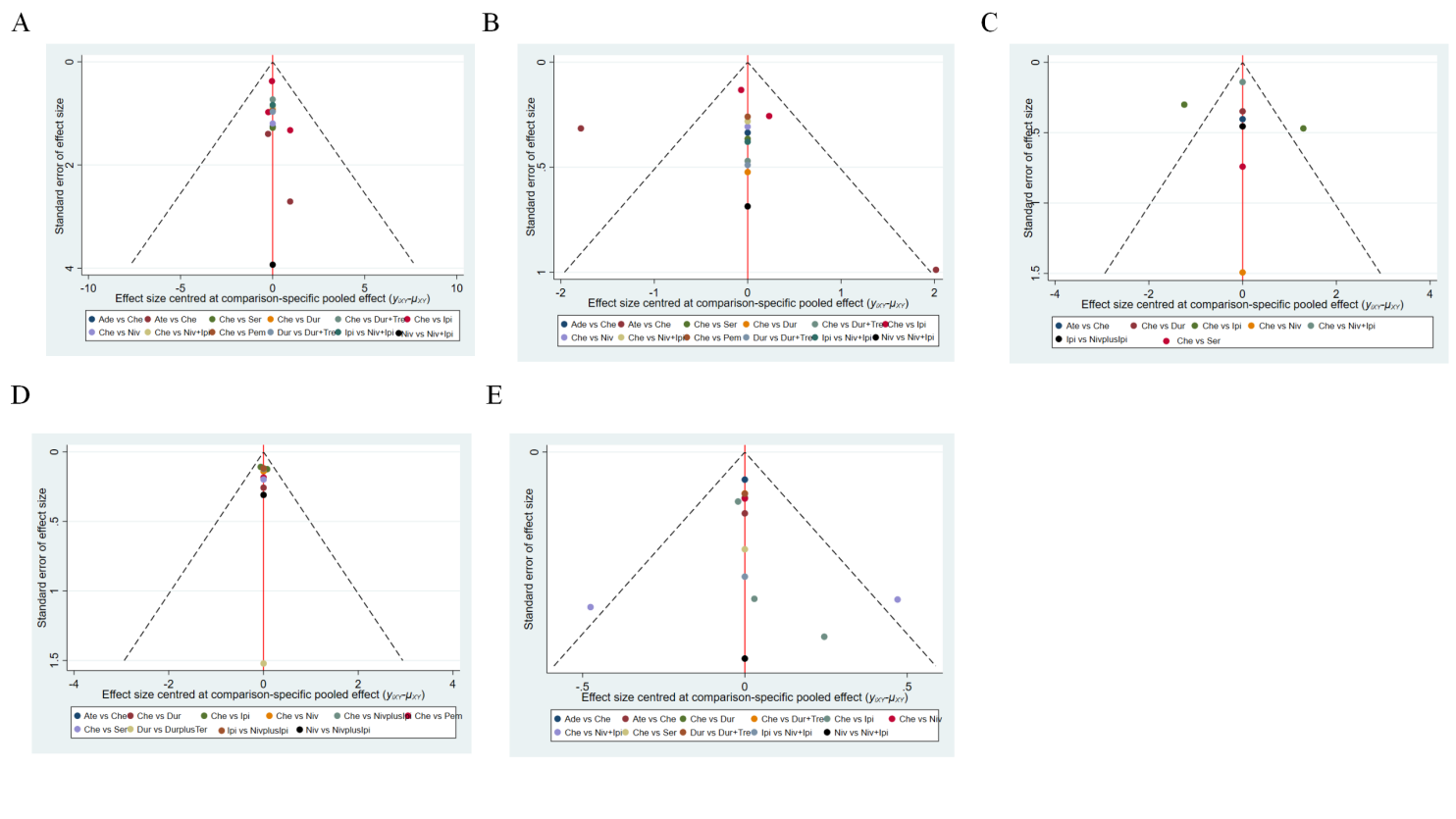
**

**Figure S5.** Funnel plot for the detection of different interventions. A: OS; B: PFS; C: DOR; D: SOR; E: AE grade ≥ 3. Che: chemotherapy; Ate: Atezolizumab; Dur: Durvalumab; DurplusTre: Durvalumab + Tremelimumab; Ipi: Ipilimumab; Pem: Pembrolizumab; Ser: Serplulimab; Niv: Nivolumab; NivplusIpi: Nivolumab + Ipilimumab; Ade: Adebrelimab. PFS: progression-free survival; OS: overall survival; DOR: duration of response; SOR: stable of response; AE grade ≥ 3: adverse events of grade ≥ 3.

**
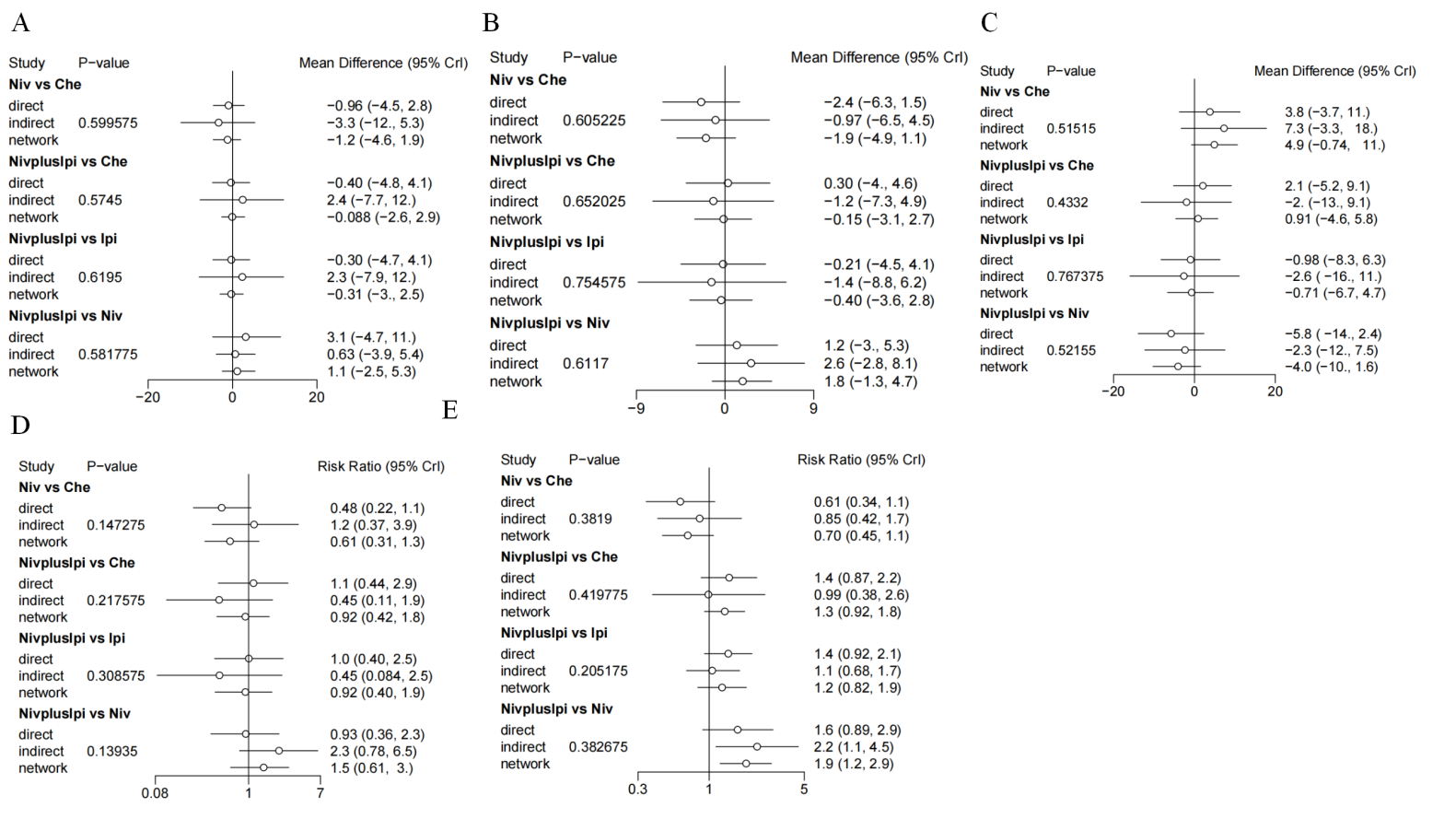
**

**Figure S6.** Node-splitting graph of different interventions. A: OS; B: PFS; C: DOR; D: SOR; E: AE grade ≥ 3. Che: chemotherapy; Ipi: Ipilimumab; Niv: Nivolumab; NivplusIpi: Nivolumab + Ipilimumab; PFS: progression-free survival; OS: overall survival; DOR: duration of response; SOR: stable of response; AE grade ≥ 3: adverse events of grade ≥ 3.

| **Table S1.** Literature search strategy. | | |
| --- | --- | --- |
| Search Strategy in PubMed | | |
| #1 | Small Cell Lung Carcinoma[MeSH Terms] | |
| #2 | small cell lung carcinoma[Title/Abstract] OR Small Cell Lung Cancer[Title/Abstract] OR bronchial small cell cancer[Title/Abstract] OR bronchial small cell carcinoma[Title/Abstract] OR lung oat cell carcinoma[Title/Abstract] OR lung small cell cancer[Title/Abstract] OR lung small cell carcinoma[Title/Abstract] OR microcellular lung carcinoma[Title/Abstract] OR oat cell cancer of the lung[Title/Abstract] OR oat cell carcinoma of the lung[Title/Abstract] OR oat cell lung cancer[Title/Abstract] OR oat cell lung carcinoma[Title/Abstract] OR pulmonary small cell cancer[Title/Abstract] OR pulmonary small cell carcinoma[Title/Abstract] OR small cell bronchial cancer[Title/Abstract] OR small cell bronchial carcinoma[Title/Abstract] OR small cell cancer of the lung[Title/Abstract] OR small cell carcinoma of the lung[Title/Abstract] OR small cell lung tumor[Title/Abstract] OR small cell neuroendocrine carcinoma of the lung[Title/Abstract] OR small cell pulmonary cancer[Title/Abstract] OR small cell pulmonary carcinoma[Title/Abstract] | |
| #3 | #1 OR #2 | |
| #4 | Anti-CTLA-4 MAb Ipilimumab[Title/Abstract] OR Anti CTLA 4 MAb Ipilimumab[Title/Abstract] OR Yervoy[Title/Abstract] OR MDX 010[Title/Abstract] OR MDX010[Title/Abstract] OR MDX-010[Title/Abstract] OR MDX-CTLA-4[Title/Abstract] OR MDX CTLA 4[Title/Abstract] OR bms 734016[Title/Abstract] OR bms734016[Title/Abstract] OR cs 1002[Title/Abstract] OR cs1002[Title/Abstract] OR eb 1003[Title/Abstract] OR eb1003[Title/Abstract] OR hlx 13[Title/Abstract] OR hlx13[Title/Abstract] OR ibi 310[Title/Abstract] OR ibi310[Title/Abstract] OR mdx 010[Title/Abstract] OR mdx 101[Title/Abstract] OR mdx010[Title/Abstract] OR mdx101[Title/Abstract] OR pbp 1701[Title/Abstract] OR pbp1701[Title/Abstract] OR strentarga[Title/Abstract] OR yervoy[Title/Abstract] OR ipilimumab[Title/Abstract] OR MPDL3280A[Title/Abstract] OR MPDL-3280A[Title/Abstract] OR Tecentriq[Title/Abstract] OR RG7446[Title/Abstract] OR RG-7446[Title/Abstract] OR anti-PDL1[Title/Abstract] OR monoclonal antibody mpdl 3280a[Title/Abstract] OR monoclonal antibody mpdl3280a[Title/Abstract] OR mpdl 3280a[Title/Abstract] OR mpdl3280a[Title/Abstract] OR rg 7446[Title/Abstract] OR rg7446[Title/Abstract] OR ro 5541267[Title/Abstract] OR ro5541267[Title/Abstract] OR tecentriq[Title/Abstract] OR tecntriq[Title/Abstract] OR atezolizumab[Title/Abstract] OR MEDI4736[Title/Abstract] OR MEDI-4736[Title/Abstract] OR Imfinzi[Title/Abstract] OR imfinzi[Title/Abstract] OR medi 4736[Title/Abstract] OR medi4736[Title/Abstract] OR durvalumab[Title/Abstract] OR SCH-900475[Title/Abstract] OR lambrolizumab[Title/Abstract] OR MK-3475[Title/Abstract] OR Keytruda[Title/Abstract] OR bcd 201[Title/Abstract] OR bcd201[Title/Abstract] OR keytruda[Title/Abstract] OR lambrolizumab[Title/Abstract] OR mk 3475[Title/Abstract] OR mk3475[Title/Abstract] OR pbp 2102[Title/Abstract] OR pbp2102[Title/Abstract] OR sch 900475[Title/Abstract] OR sch900475[Title/Abstract] OR xtrudane[Title/Abstract] OR pembrolizumab[Title/Abstract] OR adebrelimab[Title/Abstract] OR hansizhuang[Title/Abstract] OR hlx 10[Title/Abstract] OR hlx10[Title/Abstract] OR serplulimab[Title/Abstract] OR mtig 7192a[Title/Abstract] OR mtig7192a[Title/Abstract] OR ro 7092284[Title/Abstract] OR ro7092284[Title/Abstract] OR tiragolumab[Title/Abstract] OR Opdivo[Title/Abstract] OR ONO-4538[Title/Abstract] OR ONO 4538[Title/Abstract] OR ONO4538[Title/Abstract] OR MDX-1106[Title/Abstract] OR MDX 1106[Title/Abstract] OR MDX1106[Title/Abstract] OR BMS-936558[Title/Abstract] OR BMS 936558[Title/Abstract] OR BMS936558[Title/Abstract] OR ba 1104[Title/Abstract] OR ba1104[Title/Abstract] OR bms 936558[Title/Abstract] OR bms936558[Title/Abstract] OR cmab 819[Title/Abstract] OR cmab819[Title/Abstract] OR ly 01015[Title/Abstract] OR ly01015[Title/Abstract] OR mdx 1106[Title/Abstract] OR mdx1106[Title/Abstract] OR ono 4538[Title/Abstract] OR ono4538[Title/Abstract] OR opdivo[Title/Abstract] OR pbp 2101[Title/Abstract] OR pbp2101[Title/Abstract] OR xdivane[Title/Abstract] OR nivolumab[Title/Abstract] | |
| #5 | randomized controlled trial[Title/Abstract] OR RCT[Title/Abstract] OR controlled clinical trial[Title/Abstract] OR randomized[Title/Abstract] OR randomly[Title/Abstract] OR trial[Title/Abstract] | |
| #6 | #3 AND #4 AND #5 | |
| Search Strategy in Embase | | |
| #1 | 'small cell lung cancer'/exp | |
| #2 | 'small cell lung carcinoma':ab,ti OR 'small cell lung cancer':ab,ti OR 'bronchial small cell cancer':ab,ti OR 'bronchial small cell carcinoma':ab,ti OR 'lung oat cell carcinoma':ab,ti OR 'lung small cell cancer':ab,ti OR 'lung small cell carcinoma':ab,ti OR 'microcellular lung carcinoma':ab,ti OR 'oat cell cancer of the lung':ab,ti OR 'oat cell carcinoma of the lung':ab,ti OR 'oat cell lung cancer':ab,ti OR 'oat cell lung carcinoma':ab,ti OR 'pulmonary small cell cancer':ab,ti OR 'pulmonary small cell carcinoma':ab,ti OR 'small cell bronchial cancer':ab,ti OR 'small cell bronchial carcinoma':ab,ti OR 'small cell cancer of the lung':ab,ti OR 'small cell carcinoma of the lung':ab,ti OR 'small cell lung tumor':ab,ti OR 'small cell neuroendocrine carcinoma of the lung':ab,ti OR 'small cell pulmonary cancer':ab,ti OR 'small cell pulmonary carcinoma':ab,ti | |
| #3 | #1 OR #2 | |
| #4 | 'anti-ctla-4 mab ipilimumab':ab,ti OR 'anti ctla 4 mab ipilimumab':ab,ti OR 'mdx-010':ab,ti OR 'mdx-ctla-4':ab,ti OR 'mdx ctla 4':ab,ti OR 'bms 734016':ab,ti OR 'bms734016':ab,ti OR 'cs 1002':ab,ti OR 'cs1002':ab,ti OR 'eb 1003':ab,ti OR 'eb1003':ab,ti OR 'hlx 13':ab,ti OR 'hlx13':ab,ti OR 'ibi 310':ab,ti OR 'ibi310':ab,ti OR 'mdx 010':ab,ti OR 'mdx 101':ab,ti OR 'mdx010':ab,ti OR 'mdx101':ab,ti OR 'pbp 1701':ab,ti OR 'pbp1701':ab,ti OR 'strentarga':ab,ti OR 'yervoy':ab,ti OR 'ipilimumab':ab,ti OR 'mpdl-3280a':ab,ti OR 'rg-7446':ab,ti OR 'anti-pdl1':ab,ti OR 'monoclonal antibody mpdl 3280a':ab,ti OR 'monoclonal antibody mpdl3280a':ab,ti OR 'mpdl 3280a':ab,ti OR 'mpdl3280a':ab,ti OR 'rg 7446':ab,ti OR 'rg7446':ab,ti OR 'ro 5541267':ab,ti OR 'ro5541267':ab,ti OR 'tecentriq':ab,ti OR 'tecntriq':ab,ti OR 'atezolizumab':ab,ti OR 'medi-4736':ab,ti OR 'imfinzi':ab,ti OR 'medi 4736':ab,ti OR 'medi4736':ab,ti OR 'durvalumab':ab,ti OR 'sch-900475':ab,ti OR 'mk-3475':ab,ti OR 'bcd 201':ab,ti OR 'bcd201':ab,ti OR 'keytruda':ab,ti OR 'lambrolizumab':ab,ti OR 'mk 3475':ab,ti OR 'mk3475':ab,ti OR 'pbp 2102':ab,ti OR 'pbp2102':ab,ti OR 'sch 900475':ab,ti OR 'sch900475':ab,ti OR 'xtrudane':ab,ti OR 'pembrolizumab':ab,ti OR 'adebrelimab':ab,ti OR 'hansizhuang':ab,ti OR 'hlx 10':ab,ti OR 'hlx10':ab,ti OR 'serplulimab':ab,ti OR 'mtig 7192a':ab,ti OR 'mtig7192a':ab,ti OR 'ro 7092284':ab,ti OR 'ro7092284':ab,ti OR 'tiragolumab':ab,ti OR 'ono-4538':ab,ti OR 'mdx-1106':ab,ti OR 'bms-936558':ab,ti OR 'ba 1104':ab,ti OR 'ba1104':ab,ti OR 'bms 936558':ab,ti OR 'bms936558':ab,ti OR 'cmab 819':ab,ti OR 'cmab819':ab,ti OR 'ly 01015':ab,ti OR 'ly01015':ab,ti OR 'mdx 1106':ab,ti OR 'mdx1106':ab,ti OR 'ono 4538':ab,ti OR 'ono4538':ab,ti OR 'opdivo':ab,ti OR 'pbp 2101':ab,ti OR 'pbp2101':ab,ti OR 'xdivane':ab,ti OR 'nivolumab':ab,ti | |
| #5 | 'randomized controlled trial':ab,ti OR 'rct':ab,ti OR 'controlled clinical trial':ab,ti OR 'randomized':ab,ti OR 'randomly':ab,ti OR 'trial':ab,ti |  |
| #6 | #3 AND #4 AND #5 |  |
| Search Strategy in Cochrane library | | |
| #1 | MeSH descriptor: [Small Cell Lung Carcinoma] explode all trees | |
| #2 | (small cell lung carcinoma OR Small Cell Lung Cancer OR bronchial small cell cancer OR bronchial small cell carcinoma OR lung oat cell carcinoma OR lung small cell cancer OR lung small cell carcinoma OR microcellular lung carcinoma OR oat cell cancer of the lung OR oat cell carcinoma of the lung OR oat cell lung cancer OR oat cell lung carcinoma OR pulmonary small cell cancer OR pulmonary small cell carcinoma OR small cell bronchial cancer OR small cell bronchial carcinoma OR small cell cancer of the lung OR small cell carcinoma of the lung OR small cell lung tumor OR small cell neuroendocrine carcinoma of the lung OR small cell pulmonary cancer OR small cell pulmonary carcinoma):ti,ab,kw | |
| #3 | #1 OR #2 | |
| #4 | (Anti-CTLA-4 MAb Ipilimumab OR Anti CTLA 4 MAb Ipilimumab OR Yervoy OR MDX 010 OR MDX010 OR MDX-010 OR MDX-CTLA-4 OR MDX CTLA 4 OR bms 734016 OR bms734016 OR cs 1002 OR cs1002 OR eb 1003 OR eb1003 OR hlx 13 OR hlx13 OR ibi 310 OR ibi310 OR mdx 010 OR mdx 101 OR mdx010 OR mdx101 OR pbp 1701 OR pbp1701 OR strentarga OR yervoy OR ipilimumab OR MPDL3280A OR MPDL-3280A OR Tecentriq OR RG7446 OR RG-7446 OR anti-PDL1 OR monoclonal antibody mpdl 3280a OR monoclonal antibody mpdl3280a OR mpdl 3280a OR mpdl3280a OR rg 7446 OR rg7446 OR ro 5541267 OR ro5541267 OR tecentriq OR tecntriq OR atezolizumab OR MEDI4736 OR MEDI-4736 OR Imfinzi OR imfinzi OR medi 4736 OR medi4736 OR durvalumab OR SCH-900475 OR lambrolizumab OR MK-3475 OR Keytruda OR bcd 201 OR bcd201 OR keytruda OR lambrolizumab OR mk 3475 OR mk3475 OR pbp 2102 OR pbp2102 OR sch 900475 OR sch900475 OR xtrudane OR pembrolizumab OR adebrelimab OR hansizhuang OR hlx 10 OR hlx10 OR serplulimab OR mtig 7192a OR mtig7192a OR ro 7092284 OR ro7092284 OR tiragolumab OR Opdivo OR ONO-4538 OR ONO 4538 OR ONO4538 OR MDX-1106 OR MDX 1106 OR MDX1106 OR BMS-936558 OR BMS 936558 OR BMS936558 OR ba 1104 OR ba1104 OR bms 936558 OR bms936558 OR cmab 819 OR cmab819 OR ly 01015 OR ly01015 OR mdx 1106 OR mdx1106 OR ono 4538 OR ono4538 OR opdivo OR pbp 2101 OR pbp2101 OR xdivane OR nivolumab):ti,ab,kw | |
| #5 | (randomized controlled trial OR RCT OR controlled clinical trial OR randomized OR randomly OR trial):ti,ab,kw | |
| #6 | #3 AND #4 AND #5 | |
| Search Strategy in CNKI | | |
| #1 | 篇关摘：小细胞肺癌 | |
| #2 | 篇关摘：伊匹木单抗 + 阿替利珠单抗 + 度伐利尤单抗 + 度伐利尤单抗联合伊匹木单抗 + 帕博利珠单抗 + 阿得贝利单抗 + 斯鲁利单抗 + 阿替利珠单抗联合替瑞利尤单抗 + 纳武利尤单抗 | |
| #3 | 随机对照 + RCT + 对照 + 随机 + 临床研究 + 临床观察 | |
| #4 | #1 AND #2 AND #3 | |
| Search Strategy inWanfang database | | |
| #1 | 主题：小细胞肺癌 | |
| #2 | 主题：伊匹木单抗 OR 阿替利珠单抗 OR 度伐利尤单抗 OR 度伐利尤单抗联合伊匹木单抗 OR 帕博利珠单抗 OR 阿得贝利单抗 OR 斯鲁利单抗 OR 阿替利珠单抗联合替瑞利尤单抗 OR 纳武利尤单抗 | |
| #3 | 主题：随机对照 OR RCT OR 对照 OR 随机 OR 临床研究 OR 临床观察 | |
| #4 | #1 AND #2 AND #3 | |

**Table S2.** Baseline Clinical and disease Characteristics of Trials.

|  | Age | | smoke | | ECOG performance-status score | | Brain metastases at enrollment | | Liver metastases at enrollment | | PD-L1 tumour proportion score | | | Race | | | n |
| --- | --- | --- | --- | --- | --- | --- | --- | --- | --- | --- | --- | --- | --- | --- | --- | --- | --- |
|  | <65 | ≥65 | Never | Current  /former | 0 | 1 or 2 | Yes | No | Yes | No | <1% | ≥1% | Not evaluable | Asian | White | other |  |
| Reck M,et al. 2013 | 99 | 31 | 14 | 116 | 31 | 99 | NR | NR | NR | NR | NR | NR | NR | NR | NR | NR | 130 |
| Paz-Ares L,et al.2019-2022 | 478 | 327 | 52 | 753 | NR | NR | 93 | 712 | 329 | 476 | NR | NR | NR | 125 | 665 | 15 | 805 |
| Wang J,et al. 2022 | 302 | 160 | 103 | 359 | 63 | 399 | 10 | 452 | 147 | 315 | 396 | 44 | 22 | NR | NR | NR | 462 |
| Peters S,et al. 2022 | 63 | 90 | 1 | 152 | 48 | 105 | NR | NR | NR | NR | NR | NR | NR | NR | NR | NR | 153 |
| Ready NE,et al. 2020 | 129 | 114 | 14 | 233 | 76 | 167 | NR | NR | 112 | 131 | NR | NR | NR | NR | NR | NR | 243 |
| Cheng Y,et al. 2022 | 354 | 231 | 116 | 469 | 103 | 482 | 78 | 507 | 150 | 435 | 469 | 96 | NR | 401 | 184 | | 585 |
| Rudin CM,et al. 2020 | 216 | 237 | 16 | 437 | 116 | 337 | 55 | 398 | 187 | 266 | 175 | 185 | 93 | NR | NR | NR | 453 |
| Owonikoko TK,et al.2021 | 423 | 411 | 53 | 781 | 332 | 502 | 116 | 718 | 325 | 509 | 191 | 163 | 480 | 185 | 627 | 22 | 834 |
| Reck M,et al. 2016 | 576 | 378 | 56 | 898 | 284 | 670 | 100 | 854 | NR | NR | NR | NR | NR | 215 | 724 | 15 | 954 |
| Spigel DR,et al. 2021 | 361 | 208 | 50 | 519 | 156 | 413 | 96 | 473 | 205 | 364 | 175 | 146 | NR | 141 | 422 | 6 | 569 |
| Antonia SJ,et al.2019 | 143 | 16 | 7 | 152 | NR | NR | NR | NR | NR | NR | 87 | NR | NR | NR | 151 | 8 | 159 |
| Horn L,et al. 2018 | 217 | 186 | 12 | 391 | 140 | 263 | 35 | 368 | 149 | 254 | NR | NR | NR | NR | NR | NR | 403 |

Table S3. Incidence of Grade≥3 Adverse Events in Each Immunotherapy Combination.

| Incidence of Grade≥3 Adverse Events in Each Immunotherapy Combination(%). | | | | | | | | | | |
| --- | --- | --- | --- | --- | --- | --- | --- | --- | --- | --- |
|  | Ser | Dur | Ade | Ate | Pem | Ipi | Che | Durp+Tre | Niv+Ipi | Niv |
| Alopecia | NR | 1.13% | 0.00% | 0.00% | 0.00% | 0.00% | 0.28% | 0.38% | NR | NR |
| Leukopenia | 2.31% | NR | 1.13% | 5.05% | NR | 1.46% | 5.30% | NR | 11.66% | 4.16% |
| Neutrophil count decreased | 9.00% | 6.42% | 75.65% | 14.14% | NR | 0.00% | 19.99% | 4.14% | 0.00% | 0.00% |
| Platelet count decreased | 9.25% | 1.51% | 38.26% | 3.54% | NR | 1.67% | 8.89% | 1.13% | NR | 0.35% |
| Decreased white-cell count | 8.48% | 1.51% | 46.09% | 3.03% | NR | 1.26% | 9.99% | 3.01% | NR | 0.35% |
| Vomiting | NR | 0.00% | 0.87% | 1.01% | 0.90% | 1.05% | 0.95% | 1.50% | 0.88% | 0.41% |
| Asthenia | NR | 1.89% | 0.43% | NR | 3.59% | NR | 3.25% | 1.88% | 0.00% | 0.47% |
| Neutropenia | 2.57% | 24.15% | NR | 23.23% | 43.50% | 16.73% | 25.39% | 31.95% | NR | 0.35% |
| Rash | NR | NR | NR | NR | 1.35% | 1.22% | 0.08% | 1.13% | 1.45% | 1.22% |
| cough | NR | 0.75% | NR | NR | 0.45% | NR | 0.35% | 0.38% | 0.00% | NR |
